# Campaign Governance and Playfulness: Unraveling Chinese HPV Immunization Promotion Efforts on Douyin

**DOI:** 10.3390/healthcare12161657

**Published:** 2024-08-20

**Authors:** Jiaji Wu, Ronghui Yang

**Affiliations:** College of Humanities, Donghua University, Shanghai 200051, China; jiajiwu@dhu.edu.cn

**Keywords:** HPV vaccine, campaign governance, playfulness, Douyin

## Abstract

(1) Background: Playful immunization promotion helps to approach persuasive efforts and raise vaccine acceptance. However, playful promotion in the field of HPV immunization has not been explored. (2) Methods: To address this gap, we analyzed data gleaned from 73 short videos posted by state media and from semi-structured interviews with 37 Chinese stakeholders using thematic analysis. (3) Results: The analysis revealed that state media promoted HPV immunization using celebrity endorsement, anthropomorphism techniques and entertainment performance strategies. Additionally, state media engaged in circle mobilization and livestreamed on Douyin to reach wider audiences. Although playful strategies increased the popularity of HPV vaccine promotion, insufficient multi-stakeholder partnerships and homogeneous message delivery decreased the efficiency of HPV immunization promotion campaigns. (4) Conclusions: The strengthening of multi-stakeholder partnerships and the optimization of the public service provision of HPV vaccination are expected. Our research will not only deepen the global audience’s understanding of Chinese immunization promotion campaigns, but also offer insights for implementing future global health promotion.

## 1. Introduction

Cervical cancer, considered the most common gynecological malignancy in the female reproductive tract, poses a serious threat to women ‘s health worldwide. HPV vaccinations play a critical role in preventing HPV infection. In November 2020, the World Health Organization officially launched its global strategy for HPV vaccination. The strategy is intended to achieve several targets by 2030, including vaccinating 90% of girls with the human papillomavirus (HPV) vaccine by the age of 15. These goals have become targets for all 194 participating countries to work toward [1]. Therefore, countries around the world implemented HPV vaccine campaigns in 2018 to manage HPV infection. In order to provide technical, financial and policy support for implementing HPV immunization promotion efforts at the local level, the Chinese central state issued a number of policies, such as the “Key Points of Health China Initiative for 2021”, released in April 2021, and the “Outline for the Development of Chinese Women (2021–2023)”, issued in January 2019 [2].

Current research on HPV immunization in China primarily examines the context, methods and outcomes of mobilization efforts. Despite increasing vaccination rates, HPV vaccine hesitancy remains a significant issue, driven by a complex interplay of cultural, informational, and individual factors. For instance, Gao et al. (2022) highlight that many individuals, especially in rural areas, possess limited knowledge about HPV and the vaccine, leading to misunderstandings and skepticism about its necessity and benefits [3]. Li et al. (2022) emphasize that cultural attitudes towards sexual health and preventive measures critically influence vaccine acceptance [4]. Additionally, the spread of misinformation on social media exacerbates hesitancy, with common misconceptions, including exaggerated fears of side effects and doubts about the vaccine’s efficacy [5,6].

In this context, deploying a campaign-style approach for promoting HPV vaccines helps to prioritize the protection public health, overcome bureaucracy, and allocate resources to quickly achieve immunization goals [7]. Therefore, Hu et. al. demonstrate that states primarily advocate collectivism and patriotism to promote vaccine acceptance among the public [8,9]. Specially, Wang’s research illustrates that senior governments employed political incentives, established political targets for local governments, and implemented accountability efforts to encourage vaccinations [10]. Additionally, Zhang et. al. indicate that in response to public fear and anxiety during the COVID-19 crises, the Chinese government has coordinated community volunteers and enlisted prominent figures as role models [11,12]. Hu argues that Chinese government targeted initiatives have been launched to address specific demographics or regions with lower vaccine uptake by utilizing tailored educational materials designed to overcome unique cultural or logistical barriers [13]. As Zhang et. al. demonstrate, mobilization-style governance has significantly increased vaccine acceptance [14]. However, “hard” promotion backfired over the last few decades, as it does not conform to the concept of a service-oriented government raised by the central state in 2004. According to Pu et. al., hard promotion tactics that concern government values and collective values but ignore individualism induced public dissatisfaction, thereby decreasing the efficiency of promotion campaigns [15,16].

In reality, Chinese states launched extensive HPV immunization promotion campaigns (HIPCs) on social media in 2023 to raise HPV vaccine acceptance. Implementing the HIPCs via social media helps to maximize exposure to vaccine-related information and alter target audiences’ beliefs towards vaccinations in desired ways. To further confirm research lacunae, we trace academic debates on entertaining promotions on digital platforms in China. The literature primarily explores entertainment-based mobilization methods and outcomes in the field of health promotion. Wang, Ma, and Luo et al. investigated various entertaining promotions on digital platforms, including celebrity endorsements, political artistic performances, and anthropomorphism [17,18]. For instance, Lu indicated that celebrity endorsements emphasize the use of celebrities’ fame or their stories to achieve consensus mobilization [19]. Celebrities partnered with their followers, fostering a group that embraced social movement ideologies and engaged in relevant activities [20]. Chen et. al. pointed out that political artistic performances involve using a variety of artistic forms, such as nationalist songs, political films, posters, poems, novels, and slogans to romanticize the past, stimulate collective memories about political authority, and ensure public compliance, which can stimulate collective actions [21,22]. Qiang stated that anthropomorphism has been described as the extension of theory of mind to non-human agents [23]. This strategy is considered a flexible way of gaining political support [24].

Taken together, prior studies focused on playful strategies to promote healthy behavior on social media. However, the implementation of playful promotion efforts in the field of immunization has been undertaken less frequently. Additionally, previous research focused on pull strategies to attract audiences seeking brand information but did not explore push strategies for maximizing exposure to promotional messages. To address the research lacuna, we explored how digital HIPCs were implemented in China. This study will not only deepen global audiences’ understanding of digital vaccine promotion efforts in China, but it will also provide potential solutions for implementing global and local health promotion campaigns. In the following sections, we present the methods through which we achieved this and their results, and discuss the findings and our contributions to academic debates about playful HPV immunization promotion.

## 2. Materials and Methods

### 2.1. Research Design

In this study, we collected data from short videos on Douyin (the Chinese version of TikTok, launched by ByteDance in 2016) and conducted semi-structured interviews with diverse stakeholders. As of 2023, Douyin’s number of monthly active users reached 746.5 million, making it the most important platform for state-run outlets to promote vaccinations [25]. We first searched for short Douyin videos containing “HPV vaccine” (HPV疫苗), “bivalent vaccine” (二价疫苗), “quadrivalent vaccine” (四价疫苗), and “nine-valent vaccine” (九价疫苗) from October 2023 to January 2024. We then included videos posted by verified state media and excluded videos that promoted HPV vaccinations outside China and that were posted by unverified state media. Two authors of this paper with expertise in semi-structured interviews and immunization promotion conducted semi-structured interviews with Chinese stakeholders, including government officials, citizens, and health workers, to gather data in an exploratory manner. Government officials formulate policies and allocate resources; citizens, the target audience of vaccine promotion campaigns, have significant lay knowledge and experience of vaccinations; doctors play a pivotal role in administering vaccines and offering medical advice. We interviewed respondents via face-to-face interactions, as well as telephone and video calls, on WeChat between October 2023 and January 2024. Prior to the interviews, we obtained oral informed consent from participants after sharing with them the research goals, methods, expected outcomes, anticipated impacts, and their rights and responsibilities, after ensuring their anonymity.

### 2.2. Data Collection and Analysis

We obtained 73 short Douyin videos that promoted HPV immunizations. We transcribed the text from videos and created a transcript for data analysis. Qualitative data collection involved identifying, recruiting, and investigating respondents and creating an interview transcript. We first confirmed potential interviewees based on their knowledge and experiences with HIPCs on Douyin. To this end, we confirmed multiple stakeholders interviewed based on literature review, publishers of Douyin videos that promote HPV vaccinations, pre-screening interviews, and interviewers’ expertise in qualitative research and immunization promotion. We then recruited participants via random sampling and snowballing approaches in line with the specific Chinese context; selection started through informal, personal networks and continued through snowballing to include participants’ colleagues. In this way, we interviewed respondents in Anhui, Yunnan, and Shanghai, to reach data saturation. Anhui, Yunnan, and Shanghai are located in the eastern, central, and western parts of China, respectively, representing varying levels of economic development. This approach helped to ensure a diverse and representative sample of stakeholders. The research population included 19 government-official staff, 16 citizens, and 2 health workers (see Table 1). Finally, we collated the interview data and created an interview transcript using Microsoft Word 2019 for qualitative analysis.

### 2.3. Data Analysis 

Thematic analysis was employed to inductively analyze the interview transcripts, providing a comprehensive understanding of the key themes and patterns of HPV vaccination promotion on Douyin. According to Braun and Clarke (2006), thematic analysis involves generating initial codes, searching for themes, and producing a detailed report of the findings [26]. Specifically, we first generated initial coding and developed codebooks. Coder 1, served by the first author, conducted an initial manual coding of the interview transcripts using NVivo 12 software. This involved identifying and labeling significant segments of text that highlighted recurrent themes and key points. A tree node structure was created in NVivo 12 to visualize the hierarchical relationships between these codes, facilitating a clear overview of thematic connections. Coder 1 developed Codebook_1 based on this preliminary coding process. 

To enhance the reliability and validity of the coding process, Coder 2, served by the second author, coded the same set of interview records using NVivo 12 and then developed codebook_2, following the same procedure as Coder 1. To ensure the consistency and reliability of the coding process, the Kappa coefficient was calculated using NVivo 12. This statistical measure assessed the agreement between Coder 1 and Coder 2, to identify and quantify any discrepancies in coding. Secondly, Coder 3, supported by an external researcher with expertise in qualitative analysis and vaccine promotion, facilitated a discussion between Coder 1 and Coder 2 to address and resolve any coding discrepancies. Through this collaborative review, the final codebook was refined and confirmed, ensuring that all themes were accurately represented.

The thematic analysis led to the identification of several sub-themes related to HPV vaccination promotion strategies on Douyin. These sub-themes included celebrity endorsement strategies, anthropomorphism techniques, entertainment performance strategies, engaging in circle mobilization, and live streaming strategies (see Appendix A). Each sub-theme was validated by cross-referencing with the interview records to ensure accuracy and relevance. The alignment between the identified themes and the actual interview data was carefully reviewed to confirm the robustness of the findings. Finally, in the results section, exemplary data excerpts were selected to illustrate the key themes and sub-themes.

## 3. Results

In this chapter, we unfold the scope of HPV vaccine promotion, discuss the publishers of promotion videos, playful promotion strategies, video pushing tactics, and highlight the implications of digital HIPCs for Chinese health promotion efforts.

### 3.1. HIPCs on Douyin

In China, the HPV vaccines that citizens are able to access include the nine-valent and quadrivalent HPV vaccine from Merck & Co., based in Kenilworth, USA, for females aged 9 to 45, and the bivalent HPV vaccine from GlaxoSmithKline in Brentford, UK, and from Wantai Bio in Beijing, China, for females aged 9 to 45, as well as from Watson Bio in Shanghai, China, for females aged 9 to 30. HPV vaccinations are typically administered at local community hospitals or community health centers, with some larger hospitals offering these services through their gynecology departments. Vaccine availability can vary between Chinese cities. Currently, the HPV vaccine is not provided free of charge and is generally not covered by basic medical insurance. Nevertheless, certain local policies or public health programs may offer funding or subsidies for the vaccine, with such provisions varying by region. To promote HPV vaccinations, the central government rolled out HPV immunization campaigns and issued the “Outline for the Development of Chinese Women (2021–2023)” in 2023, to achieve a cervical cancer screening rate of 70% among eligible women by 2030 [27]. To increase the public awareness and acceptance of HPV vaccinations, government agencies at all levels launched large-scale vaccination promotion campaigns on Chinese social media platforms such as Douyin, Weibo, and WeChat.

On Douyin, the publishers of vaccination promotion videos were the local People’s Government, the Health Commission, the Media Convergence Center, Local Media Integration, the Communist Youth League, the education sector, and public medical institutions. The scope of HPV promotion on Douyin primarily covers topics such as vaccination-related policies, the potential side effects of HPV vaccination, and the procedures, requirements, and importance of HPV vaccination. In response, state media utilized short videos to disseminate knowledge about HPV vaccinations and refute rumors.

### 3.2. Playful Rhetoric for Promoting HPV Immunization

On Douyin, state media promoted HPV vaccinations using celebrity endorsement strategies, anthropomorphism techniques, comic strategies and entertainment performance strategies.

#### 3.2.1. Celebrity Endorsement 

Celebrity endorsement refers to the promotion of a product or an idea by public figures. Famous people have widespread impact and could raise public awareness of a social issue, which is likely to positively affect consumers’ attitudes [28]. To improve the acceptance of HPV vaccines, various celebrities, such as singers, public health experts, and stars from film and television served as vaccine ambassadors in Douyin’s HIPCs. In the HIPCs, celebrities primarily used consensus mobilization and gain–loss framing strategies to persuade the public.

Consensus mobilization centers on confirming what most appeals to the target audience and then adopting tailored tactics to convince them. Since the target audience for HPV vaccination is Chinese females, state media preferred to invite and showcase healthy female celebrities in short videos, highlighting female empowerment and the spirit of girls supporting girls to convey the value proposition of “get vaccinated, care for yourself” (ID 7). In Douyin’s HIPCs, consensus mobilization played an indispensable role in addressing vaccine hesitancy, and it involved both rational and emotional models. The rational model highlighted the use of facts, accurate evidence, and logical reasoning to create persuasive messages (ID 28–31). In the Douyin videos, female celebrities quoted statistical data and the results of clinic trials to increase the credibility of arguments, increase females’ rational perception of vaccinations, and ease vaccine concerns. For example, a female celebrity on Douyin noted the following:

*“In China, about 100,000 families suffer from HPV diseases every year, which is equivalent to one woman being diagnosed with cervical cancer every 5 min. According to the data from the National Cancer Center in 2024, there are approximately 55,700 deaths from cervical cancer each year, entailing that one woman dies of cervical cancer every 9 min”.*

Complementing the logical approach, the empathetic model also played a critical role in persuading targeted individuals to get vaccinated. Empathetic persuasion refers to understanding targeted individuals’ feelings, supporting their perspectives, and reassuring them regarding their concerns in an empathetic way to facilitate behavior changes. In the videos, the female celebrities shared their vaccination experiences, told their own stories, and expressed their feelings to connect with residents, comfort them regarding their concerns, and sincerely recommend that they receive their vaccination to prevent viruses. This approach helped to resonate with residents and construct public trust in HIPCs (ID 32–37). A female celebrity in a video noted the following:

*“Females in China still have a weak awareness of reproductive health. I don’t want to see the number of cervical cancer and gynecological diseases among women around me continue to rise in the future”.*

Gain–loss framing strategies refer to the phrasing of a statement that describes a choice or outcome in terms of its positive (*gain*) or negative (*loss*) features. In Douyin’s HIPCs, the celebrities argued that cervical cancer increased fatality rates among Chinese populations and threatened personal well-being. At the same time, the celebrities raised the public’s anxieties about the risk of illness, stressed the importance of vaccination, and used personification techniques to help the public better understand the benefits of vaccinations, ultimately persuading more people to accept the HPV vaccine. Furthermore, they highlighted the critical role of the HPV vaccine in preventing cervical cancer. A public health expert in a video noted the following:

*“Cervical cancer is a malignant tumor with high incidence in Chinese women. If some intervention measures are not taken, the trend may still rise in the future. The high-risk type of HPV infection is the main cause of cervical cancer. Cervical cancer is also the only cancer that can be prevented by vaccination”.*

(ID 23)

#### 3.2.2. Anthropomorphic Techniques

Anthropomorphic promotion entails a process of personifying media accounts and attributing specific human traits to them using colloquial language to present individuality and create relationships. Anthropomorphic vaccine promotion in Douyin’s HIPCs involves visual anthropomorphism, positive reinforcement, and empathic models.

Visual anthropomorphism focuses on employing expressive visual elements to give non-human entities human characteristics, emotions or behaviors, in order to promote a brand or a product vividly and resonate with audiences. In Douyin’s HIPCs, visual anthropomorphism entailed creating cartoon representations of non-human entities, such as the HPV virus and vaccine, and characterizing them with human facial expressions, body language, and physical movements to demonstrate the role of vaccines in preventing the virus. Personification in presentation helped the public recognize the role and importance of vaccination and assisted in persuading the public to accept the vaccine. For example, the HPV virus was characterized on Douyin as a devil with disheveled black hair and a cold smirk on its lips, suddenly appearing in front of a cute girl. It waved its vicious tentacles and roared: “*I will destroy you!*” However, the girl confidently retorted: “*I got the HPV vaccine, so I can become superwomen. I am not afraid of you, the big bad guy!*” Consequently, failing to intimidate the girl, the virus ran away in frustration.

Positive reinforcement, as coined by B.F. Skinner, refers to the principle that when the outcome of a behavior is beneficial, the behavior is likely to be repeated in the future [29]. This strategy emphasizes positive consequences or praise as a means to guide an individual’s behavior. In Douyin’s HIPCs, state media reinforced their value identification through the use of affirmative and praising verbs and consecutive exclamation words. For instance, state media proudly proclaimed in a video: “Great Achievement! It’s time to get vaccinated!” The message continued with enthusiasm: “Exciting Update! Here’s the latest: the HPV vaccinations are now available! Don’t hesitate, visit your nearest hospital and get vaccinated as soon as possible. It’s a crucial step towards protecting your health”. The video, using affirmative exclamation words and a positive tone, was intended to motivate the public and emphasize the importance of the HPV vaccine in promoting public health and preventing diseases.

Empathetic persuasion refers to gaining insights into audiences’ feelings, supporting their perspectives, and reassuring them regarding their concerns in an empathetic way to foster a sense of trust and promote behavior changes. In Douyin’s HIPCs, state media interacted with the public and responded to their comments and concerns about HPV vaccinations in comment spaces under videos using empathetic persuasion. For example, the state media engaged with users who expressed fears or doubts about the safety of HPV vaccinations. The media acknowledged these concerns and shared information from trusted medical sources to address any misconceptions. Additionally, they also shared personal stories from individuals who had positive experiences with HPV vaccinations, aiming to provide reassurance through empathy. Furthermore, the state media actively encouraged open dialogue by asking questions such as: “What are your main worries about HPV vaccinations?” This approach allowed the media to understand the specific concerns of their audiences and tailor their responses accordingly. In response, the commentaries provided by state media demonstrated a full understanding of and sympathy towards public concerns. The media patiently explained the current vaccination supply situation, offered alternative solutions, and called on individuals to maintain rationality and patience.

#### 3.2.3. Entertainment Performance 

Entertainment performance focuses on disseminating information in an entertaining way. In Douyin’s HIPVs, state media primarily used reciprocal interactions between experts and laypeople, as well as artistic performances.

Unlike the one-way information dissemination channels used in previous publicity campaigns, interactive communication focuses on the interaction between the government and the public, the efficient dissemination of information, and persuading the public to receive vaccinations. In Douyin’s HIPVs, the public raised questions and doubts, and experts responded with scientific information, such as numerical support and scientific arguments. For example, in an interview video posted by state media, parents, young people, and clinicians interacted mutually to discuss the importance of vaccination and addressed concerns or questions the public may have about HPV vaccinations. Facing public doubts about the efficacy of HPV vaccinations, a clinician argued the following:

*“The domestic HPV vaccine Xinkening provides a protection rate of up to 100% against HPV16/18-related high-grade genital precancerous lesions in women aged from 18 to 45, as well as a protection rate of 97.3% against persistent HPV16/18 infection (6 months)”.*

(ID 36)

Artistic performances are intended to enhance the awareness of HPV vaccinations through songs and comedy. “Songs” emphasizes the use of catchy melodies and beats in brief videos, with lyrics regarding vaccines to help people have fun. In a video, the performer animatedly gestured while singing rhymes with words, as in the following example:

*“If you get HPV vaccinations, you will be protected against HPV infection. If you get HPV vaccinations, you will reduce the risk of cervical cancer. If you get HPV vaccinations, you will safeguard your health. Please get a shot”.*

Comedy, in contrast to conventional large-scale theatrical works, emphasizes condensed storytelling and vibrant rhythmic and humor expressiveness. The media typically used a variety of characters to portray a range of emotions and storylines. For example, state media showed the process of the HPV virus “invading” the body using cartoon figures and animations to make the message more enjoyable and understandable.

### 3.3. Information-Pushing Strategies 

State media not only used pull promotion strategies that attract audiences and direct them to seek information, but also employed pushing promotion strategies to maximize the exposure of promotion videos. In Douyin’s HIPVs, state media primarily adopted circle mobilization and live streaming to increase the visibility of HPV vaccine promotion messages. 

#### 3.3.1. Circle Mobilization 

Circle mobilization refers to the process of stimulating members of a social group with similar social attributes or strong social connections and similar social attributes, through internal interaction, to participate in activities and enhance cohesion and identity within social circles [30].

Although one-third of men worldwide are infected with HPV and HPV vaccination is considered important for reducing the incidence of HPV-related diseases, males are unable to receive the vaccine through official channels in China. As a result, in Douyin’s HIPCs, state media promoted the HPV vaccines to groups of women ranging from 9 to 45 years old. State media focused on specific social groups, such as families with daughters and female students, highlighting family responsibilities and campus life to attract target audiences. The focus on family responsibility emphasized the importance of parents vaccinating their daughters against HPV. For example, some videos highlighted the family responsibility of parents viewing their daughters as investments in the future, eliciting vaccine acceptance among parents. The focus on campus life showcased the daily lives of female students, attracting viewers from similar social groups. For instance, state media initiated topics that are highly relevant to the lives of female students, such as “#Female University Students Did Not Miss Out” and “#Caring For Girls Health and HPV Vaccination”, to raise awareness among Chinese female students about the necessity and importance of, and precautions surrounding HPV vaccinations. This stimulated discussions on HPV vaccination in Douyin’s comment sections and video sharing among users. A female student we interviewed stated the following:

*“I once saw a short video on Douyin featuring a female student discussing her recent dilemma about whether or not to get vaccinated for HPV. The video felt very authentic, as if it was happening around us. This immediately caught my interest. I even shared the video with other classmates”.*

(ID 19)

#### 3.3.2. Live Streaming

Live streaming is a real-time audio and video transmission system that sends live images and sounds to viewers via the internet. Live streaming played an indispensable role in promoting HPV vaccines. Live streaming offers a high degree of interaction, giving viewers a forum to discuss and seek clarification on any doubts regarding the HPV vaccine that they may have. It helps to improve vaccination willingness and eradicate public vaccine reluctance through expert explanations, case sharing, and direct interaction. In Douyin’s HIPCs, to increase audience engagement and trust, state actors responded to citizens’ inquires via live streaming. This helped audiences to gain knowledge and understand new information, and it enhanced their participation. The core of these reciprocal interactions lay in the exchange and feedback between both parties. This meant that the participants not only provided information or help in the communication process, but also expected corresponding responses or returns.

This bidirectional aspect promotes the flow and sharing of information, deepening understanding and connections between each party. For instance, the anchor in a local Chinese government live-streaming studio used the live broadcast room to respond in real time to user comments while popularizing the 9-valent vaccine, which helped to allay public fears about the vaccine. A citizen we interviewed indicated that “*during the live stream, I can ask the anchor any questions during the show*”. (ID 21)

### 3.4. Implications for Chinese Health Promotion Efforts

The HIPCs had a subtle impact on China’s health promotion efforts. The playful HPV vaccine promotion efforts on Douyin aligned with digital culture and public discourse, softened government discourse, and fostered reciprocal interaction. However, homogeneous information spread and insufficient public–private cooperation reduced the efficiency of HPV vaccine promotion on Douyin.

The HIPCs aligned with digital culture and public discourses, including the values of digitization and public dialogue. First, the innovative content formats, diverse content creation, visual effects, and optimized sound effects in the state media’s entertainment-oriented promotions quickly captured users’ attention, allowing them to easily access a large amount of information in a short period of time. Secondly, users could interact with state media through likes, comments, shares, etc., increasing engagement and sense of belonging while facilitating rapid and widespread content dissemination. Lastly, the HIPCs catered to the public’s fragmented reading habits, diverse sources of information, and interesting digital cultures (ID 3–5).

The HIPCs have adopted a more approachable government discourse. The government conventionally used authoritative strategies for propaganda, such as preaching and political mobilization. However, in Douyin’s HIPCs, the vaccine promotion campaign has shifted towards using celebrity endorsements, personification mobilization, and entertainment performances as promotional strategies. This has softened the government’s discourse in its vaccine promotion campaigns and aligned with public discourse, increasing the campaigns’ appeal to the public. As a result, the government has gradually moved from using language that is overly authoritative and serious to a more straightforward and vivid expression that avoids being didactic (ID 1).

The HIPCs fostered reciprocal interactions. The state media and the public were no longer engaged in one-way communication, but rather interacted with each other. This interaction was mainly seen in the timely and accurate responses between state media and the public. Timely responses meant being able to react or respond as quickly as possible upon receiving information, requests, or notifications. This was important for maintaining good communication and collaboration and improving efficiency. In the HIPCs, timely communication between state media and the public could be observed in the interactions with the comment spaces under videos and live streams. For example, during a live stream, when a member of the public asked about “the procedure for getting an HPV vaccine”, the state media immediately provided an answer. Accurate interactions ensured that information, data or communication during interactions were precise. All the parties involved needed to accurately understand, convey, and provide feedback to avoid misunderstandings or errors. Measures such as using precise language and terminology should have been taken to achieve accurate interactions; regularly checking and verifying information accuracy was also essential. For instance, state media provided detailed vaccination locations, including hospital opening hours and rest times, and periodically verified this information.

Furthermore, the homogenized delivery of information reduced the efficiency of the vaccine promotion campaigns. The homogenization of content is characterized by the proliferation of similar or repetitive material. Presently, the majority of short videos pertaining to HPV vaccines on Douyin are centered around themes such as “the benefits of HPV vaccines” or “information about HPV vaccine administration”, resulting in a uniformity of content. This was attributed to a lack of innovation and independent thought in news reporting by state media outlets, which tended to imitate or replicate content from other sources. The adverse effects stemming from this homogeneity in information transmission hindered comprehensive exploration and truthful news reporting, impeding public access to comprehensive vaccination information. Consequently, this diminished the efficacy of digital HIPCs by inundating the public with excessive information and complicating their ability to systematically gather vaccine-related data, ultimately leading to ineffective dissemination. A citizen we interviewed stated the following:

*“Many state media delivered similar HPV vaccine related messages, focusing mainly on the risks of not vaccinating and the benefits of vaccination. I actually want to know more vaccine-related information, such as what preparations should be made before vaccination, what should be paid attention to after vaccination, and whether there are any side effects”.*

(ID 22)

However, insufficient collaboration between the government and hospital doctors and opinion leaders in this campaign decreased the efficiency of this vaccine promotion campaign. By partnering with hospital doctors and key opinion leaders (KOLs), the government could leverage resources such as information, technology, and funding to enhance efficiency, ensure the broader dissemination of vaccine information among the public, and improve public awareness and acceptance of vaccinations. However, in terms of information dissemination strategies, there was a lack of collaboration between the government and KOLs for wider outreach. Additionally, due to insufficient collaboration with hospital doctors on content creation, some vaccine administration information was not delivered promptly. A health worker argued the following:

*“Sometimes the information published by the government on Douyin is not completely synchronized with our community. For example, sometimes our community lacks vaccines, but the government does not update this information in time and still encourages everyone to come and get vaccinated. Many people who saw the video on Douyin will come to our community and want to get vaccinated, but they will be disappointed when they find out that we don’t have any vaccines. Some people may misunderstand and blame our community hospital, saying that it has wasted their time”.*

(ID 36)

To sustain HPV vaccine promotion on media platforms, state media should set standards to censor content, and foster shared values among stakeholders for effective collaboration. In response, partnerships among government agencies should be established to clarify roles and convey differentiated information. Additionally, higher-level authorities need to set standards for preventing vulgar dissemination.

## 4. Discussion

This study was conducted to explore how Chinese governments implemented HIPCs on social media in playful ways. The analysis indicated that state media primarily promoted HPV vaccines using celebrity endorsements, anthropomorphism techniques, and entertaining performances. To maximize the exposure of these promotion videos, state media pushed HPV-vaccine-related information via topic challenges and live streaming strategies. The playful HPV vaccine promotion efforts on Douyin aligned with digital culture and public discourse, softened government discourse, and fostered reciprocal interaction. However, homogeneous information spread and insufficient public–private cooperation reduced the efficiency of HPV vaccine promotion on Douyin. Therefore, ensuring the diversity and accuracy of information delivered in vaccine campaigns should be explored in future research.

The implementation of global HPV immunization promotion efforts has directed scholarly attention to the playful HPV vaccine promotions on media platforms outside China. Extant research primarily focused on various strategies for producing entertaining promotion videos. For instance, Kim J highlighted the generation of playful promotional videos of HPV vaccines in South Korea using narrative formats, gain–loss framing, emotional appeals, and message sensation value [31]. Narrative formats are distinguished by their utilization of characters’ actions, dialogues, thoughts, and emotions to convey storylines. Rowena et al. posited that the utilization of diverse narrative perspectives (first-, second-, and third-person) enriches the interactivity, authenticity, and relatability of HPV vaccine promotion [32]. Basch proposed that promotional campaigns for HPV vaccines on TikTok may employ message framing strategies [33]. Sundstrom et al. indicated that employing loss-framed messages (emphasizing the potential risks of not receiving vaccination) is more efficacious in promoting public willingness to receive the HPV vaccine compared to gain-framed messages (emphasizing the benefits of vaccination) [34]. Similarly to previous studies, our research delved into framing messages and effectance motivation or HPV vaccination mobilization on media platforms. Building upon previous studies, this study revealed anthropomorphism strategies and information-pushing strategies in media, as well as their implications for Chinese health promotion efforts.

This study underlines playful strategies in vaccination promotion efforts in China, in contrast to the marketing mix strategies widely applied in vaccine promotion campaigns in the West. The marketing mix strategy is supported by the 7P framework, including Product, Price, Promotion, Place, People, Physical Evidence, and Process [35]. In Product management, various vaccine types should be provided according to age groups or health conditions. Regarding Price, a rational pricing strategy considering vaccine costs and market demand is needed. For government-provided free or subsidized vaccines, the focus should be on public welfare and the vaccines’ accessibility. In Promotion, channels like advertising, social media, and public speaking can be utilized for vaccine promotion and advocacy. Concerning People, healthcare professionals are encouraged to actively engage in vaccine promotion and education to enhance public awareness and acceptance. For Physical Evidence, a clear signature at vaccination sites enables the public to find them easily and display relevant information. The Process aspect involves establishing appointment systems to avoid crowding and long waiting times and to ensure effective communication between healthcare workers and the public to address inquiries and concerns about vaccinations [36]. The playful HPV vaccine promotion efforts on Douyin primarily leveraged entertainment as a mode of communication. This method is designed to seamlessly integrate brands into users’ entertainment experiences through compelling storytelling, character development, interactive engagement, and user experience, thereby augmenting brand awareness, image, and effectiveness. In contrast to marketing mix strategies that assess outcomes based on metrics such as product sales, market share, and customer satisfaction, entertainment marketing emphasizes emotional resonance by evoking users’ emotional identification with and affinity towards brands through stories and characters. It also underscores interactivity and involvement by deepening users’ comprehension of brands through active participation in activities. Moreover, it engenders a lasting impact capable of sustainably influencing and upholding a brand’s image and reputation over time. Considering its benefits, playful vaccine promotion should probably be advocated in the Western contexts. 

## 5. Limitations of the Research

This study has deepened our understanding of vaccination mobilization on new media platforms, but its limitations should be considered. First, unlike in a scientific sampling method, we selected participants through informal and snowball sampling methods in line with China’s highly relationship-oriented society, and ensured that collecting the data in this way followed the principle of data saturation. Secondly, this study focuses on stated-oriented vaccine promotion. However, non-state-actor-led HPV vaccine promotion was not explored. Therefore, future studies should focus on HPV vaccine promotion led by non-state actors and multi-stakeholder collaboration on HPV vaccine promotion.

## 6. Conclusions

On media platforms, the government’s campaign for HPV vaccinations is driven by entertainment, specifically manifested in the use of celebrity endorsements, anthropomorphic techniques, and entertaining performances to achieve content entertainment in short videos. Through engaging in circle mobilization and live streaming, the entertainment-oriented dissemination of short videos is achieved, which effectively improves the acceptance of HPV vaccination. However, homogeneous information and insufficient cooperation among stakeholders undermines the effectiveness of entertainment strategies. To address these challenges, the government needs to improve the quality of short videos, establish an adequate network of health worker partnerships for HPV vaccinations, and optimize HPV vaccination services to ensure robust promotion campaigns in modern China. 

## Figures and Tables

**Table 1:** Participants’ demographic characteristics.

Participant ID	Description of Function	Gender	Location
ID1	Staff in Anji People’s Government	Female	Huzhou
ID2	Staff in Anji People’s Government	Male	Huzhou
ID3	Staff in Anji People’s Government	Female	Huzhou
ID4	Staff in Anji People’s Government	Female	Huzhou
ID5	Staff in Anji People’s Government	Female	Huzhou
ID6	Staff in Huangpu People’s Government	Female	Shanghai
ID7	Staff in Deqing Health Commission	Male	Huzhou
ID8	Staff in Deqing Health Commission	Female	Huzhou
ID9	Staff in XuanzhouMedia Converged Center	Female	Xuancheng
ID10	Staff in Anji Media Converged Center	Female	Huzhou
ID11	Staff in Tongling Local News Center	Male	Tongling
ID12	Staff in Tongling News Center	Female	Tongling
ID13	Staff in Xuancheng Communist Youth League	Female	Xuancheng
ID14	Staff in Xuancheng Communist Youth League	Male	Xuancheng
ID15	Staff in Xuancheng Communist Youth League	Female	Xuancheng
ID16	Staff in Xuancheng Communist Youth League	Female	Hefei
ID17	Staff in Xuancheng Communist Youth League	Male	Hefei
ID18	Staff in Xuanzhuo public medical institutions	Male	Xuancheng
ID19	Douyin user	Female	Huzhou
ID20	Douyin user	Female	Huzhou
ID21	Douyin user	Female	Huzhou
ID22	Douyin user	Female	Huzhou
ID23	Douyin user	Female	Huzhou
ID24	Douyin user	Male	Huzhou
ID25	Douyin user	Male	Huzhou
ID26	Douyin user	Female	Xuancheng
ID27	Douyin user	Female	Xuancheng
ID28	Douyin user	Male	Xuancheng
ID29	Douyin user	Male	Xuancheng
ID30	Douyin user	Male	Hefei
ID31	Douyin user	Male	Hefei
ID32	Douyin user	Male	Hefei
ID33	Douyin user	Male	Hefei
ID34	Douyin user	Male	Hefei
ID35	Douyin user	Female	Shanghai
ID36	Gynecologist in a Renji private hospital	Female	Xuancheng
ID37	Gynecologist in a Renji private hospital	Female	Xuancheng

## Data Availability

The data presented in this study are available on request from the corresponding author.

## Appendix A. The Process of the Thematic Analysis

**Text (Parts of Text Are Presented Here)**	**Code**	**Sub-Theme**	**Theme**
Females in China still have a weak awareness of reproductive health. I don’t want to see the number of cervical cancer and gynecological diseases among women around me continue to rise in the future.	Celebrities’ consensus mobilization	Celebrity endorsement	Playful rhetoric for promoting HPV Immunization
Cervical cancer is a malignant tumor with high incidence in Chinese women. If some intervention measures are not taken, the trend may still rise in the future. The high-risk type of HPV infection is the main cause of cervical cancer. Cervical cancer is also the only cancer that can be prevented by vaccination.	Celebrities’ gain–loss framing		
On the Douyin platform, we will use animation styles or cartoon characters to explain complex medical concepts, thereby translating scientific information into visual forms that are easier for the audience to understand.	Visual anthropomorphism	Anthropomorphic techniques	
The HPV virus was characterized in Douyin as a devil with disheveled black hair and a cold smirk on its lips, suddenly appeared in front of a cute girl. It waved its vicious tentacles and roared: “I will destroy you!”			
Great Achievement! It’s time to get vaccinated!” The message continued with enthusiasm: “Exciting Update! Here’s the latest: the HPV vaccinations are now available! Don’t hesitate, visit your nearest hospital and get vaccinated as soon as possible. It’s a crucial step towards protecting your health”.	Positive reinforcement		
The state media actively encouraged open dialogue by asking questions such as the following: “What are your main worries about HPV vaccinations?”	Empathic models		
Facing public doubts about the efficacy of HPV vaccinations, a clinician argued the following: “The domestic HPV vaccine, Xinkening, provides a protection rate of up to 100% against HPV16/18-related high-grade genital precancerous lesions in women aged from 18 to 45, as well as a protection rate of 97.3% against persistent HPV16/18 infection (6 months)”.	Reciprocal interaction between experts and laypeople	Entertainment performance	
If you get HPV vaccinations, you will be protected against HPV infection. If you get HPV vaccinations, you will reduce the risk of cervical cancer. If you get HPV vaccinations, you will safeguard your health. Please get a shot.	Artistic performances		
As a mother of a daughter, I am highly concerned with the official media’s promotion of the HPV vaccine for adolescent girls.	Family responsibilities	Circle mobilization	Information-pushing strategies
I once saw a short video on Douyin featuring a female student discussing her recent dilemma about whether or not to get vaccinated for HPV. The video felt very authentic, as if it was happening around us. This immediately caught my interest. I even shared the video with other classmates.	Campus life		
During the live stream, I can ask the anchor any questions during the show.	High degree of interaction	Live streaming	
Douyin users can interact with state media through likes, comments, shares, etc., increasing user engagement and sense of belonging while facilitating rapid and widespread content dissemination.	Digital culture and public discourse	Positive implications	Implications for Chinese health promotion efforts
The government has gradually moved from using language that is overly authoritative and serious to a more straightforward and vivid expression that avoids being didactic.	Softened government discourse		
State media provides detailed vaccination locations, including hospital opening hours and rest times, and periodically verifies this information.	Fostered reciprocal interaction		
Many state media delivered similar HPV vaccine related messages, focusing mainly on the risks of not vaccinating and the benefits of vaccination. I actually want to know more vaccine-related information, such as what preparations should be made before vaccination, what should be paid attention to after vaccination, and whether there are any side effects.	Homogeneous information spread	Negative implications	
Sometimes the information published by the government on Douyin is not completely synchronized with our community. For example, sometimes our community lacks vaccines, but the government does not update this information in time and still encourages everyone to come and get vaccinated. Many people who saw the video on Douyin will come to our community and want to get vaccinated, but they will be disappointed when they find out that we don’t have any vaccines. Some people may misunderstand and blame our community hospital, saying that it has wasted their time.	Insufficient public–private cooperation		

## References

[1] (2018). Cervical Cancer: An NCD We Can Overcome.

[2] Liu H.W. (2021). 100 years of development of women’s rights in China: From strong political commitment to increasingly perfect legal protection. Hum. Rights.

[3] Gao Y., Liu H., Wang Z. (2022). Factors Influencing HPV Vaccine Acceptance in Rural China. Int. J. Public Health.

[4] Li Q., Zhang L., Sun J. (2022). Cultural Attitudes towards HPV Vaccination in China. J. Cult. Health Stud..

[5] Liu J., Wang P., Yang Y. (2022). Understanding Vaccine Hesitancy: A Study of HPV Vaccine Knowledge in China. J. Health Educ. Res..

[6] Liu S., Zhang H., Wang X. (2024). Addressing HPV Vaccine Hesitancy through Tailored Communication. Glob. Health Perspect..

[7] Don V. (1998). Evaluating health promotion—Progress, problems and solutions. Health Promot. Int..

[8] Hu Y.S. (2011). The Realization Mechanism of a Strong State—A Study on Community Mobilization in Shanghai during the Fight against ‘SARS’. Master’s Thesis.

[9] Liu Y.Q. (2020). Historical Experience and Enlightenment of Propaganda and Ideological Work in Responding to Major Epidemics. Ideol. Educ. Res..

[10] Wang F.S. (2022). Responding to the Situation’: Can Grassroots Governments Accomplish Tasks Under Continuous Pressure?—A Case Study Based on the Promotion of COVID-19 Vaccination in Town A, City X. J. Public Adm..

[11] Zhang J., Tao X. (2017). HPV Vaccine Research Progress, Awareness and Acceptance Status. Chin. J. Clin. Obs. Gynecol..

[12] Zhang Z.A., Peng L. (2019). Hybrid Emotional Communication Mode: Research on Short Video Content Production of Mainstream Media—Taking the People’s Daily Douyin Account as an Example. J. Writ..

[13] Hu Y.M. (2021). Health Mobilization of ‘Epidemic Prevention’ Slogans: Discourse Strategies, Frameworks, and Power Structures. J. Int. Commun..

[14] Zhang Z.L. (2008). Health Communication and Society—Changes in the Discourse of Disease Prevention and Control in China over the Past Century.

[15] Pu C., Liu C.R., Zhang X., Li J. (2018). Knowledge and attitude toward HPV and its vaccines among parents of middle school student in Chengdu. Mod. Prev. Med..

[16] Jiang N., Yang C., Yu W., Luo L., Tan X., Yang L. (2022). Changes of COVID-19 knowledge, attitudes, practices and vaccination willingness among residents in Jinan, China. Front. Public Health.

[17] Wang W.Y., Lobato R. (2019). Chinese video streaming services in the context of global platform studies. Chin. J. Commun..

[18] Luo Y.A., Wang Y.M. (2020). An Analysis of the Phenomenon of Mainstream Media’s Live Streaming of Public Welfare Goods. Media.

[19] Lu J.H. (2017). An Analysis of the Star Strategy of New Mainstream Blockbusters in the Context of Popular Culture. Contemp. Cine..

[20] Li Z.L. (2013). Clustered Stars, Micro-performances, and Their Political and Cultural Effects. Contemp. Cine..

[21] Lang J.S., Shen Q.Z. (2020). Personalized Communication of Government Short Videos: Presentation and Driving Forces—An Empirical Analysis Based on Government Douyin Accounts. Journal. Writ..

[22] Chen X., Valdovinos Kaye D.B., Zeng J. (2020). PositiveEnergy Douyin: Constructing “Playful patriotism” in a Chinese short-video application. Chin. J. Commun..

[23] Qiang Y.X., Yang Y.L. (2022). Personification: Innovation of Mainstream Media News Short Video Communication Strategy. Future Commun..

[24] Mattingly D.C., Yao E. (2022). How Soft Propaganda Persuades. Comp. Political Stud..

[25] China Internet Network Information Center China Internet Network Development State Statistic Report [EB/OL]. https://cnnic.cn/n4/2023/0302/c199-10755.html.

[26] Braun V., Clarke V. (2006). Using Thematic Analysis in Psychology. Qual. Res. Psychol..

[27] National Health Commission of China: “Accelerated Action Plan for Cervical Cancer Elimination (2022–2030). https://www.gov.cn/zhengce/zhengceku/2023-01/21/content_5738364.htm.

[28] Yang W. (2018). Star power: The evolution of celebrity endorsement research. Int. J. Contemp. Hosp. Manag..

[29] Skinner B.F. (1938). The Behavior of Organisms: An Experimental Analysis.

[30] Peng L. (2019). Circles on the Internei: Aggregation and Separation on the Dimensions of Realtionship, Culure and Technology. Ed. Friend.

[31] Kim J., Lee J., Heo J., Baek J. (2021). Message strategies and viewer responses: Content analysis of HPV vaccination videos on Youtube. J. Health Commun..

[32] Rowena B.R., Nan X.L., Kelly M., Leah W. (2012). When Vaccines Go Viral: An Analysis of HPV Vaccine Coverage on YouTube. Health Commun..

[33] Basch C.H., Hillyer G.C., Jacques E.T. (2022). Professionally Created Content Related to HPV Vaccination on TikTok. Front. Digit. Health.

[34] Sundstrom B., Cartmell K.B., White A.A., Well H., Pierce J.Y., Brandt H.M. (2021). Correcting HPV vaccination misinformation online: Evaluating the HPV vaccination NOW social media campaign. Vaccines.

[35] Xu J.Y., Wu M.Z. (2020). Analysis of Short Video Marketing Mix Strategy in Mobile Social Era. Times Ecnonomy Trade.

[36] Wasan P.G., Trpathi G. (2014). Revisting social marketing mix: A socio-cultural perspective. J. Serv. Res..

